# Characterization of Four Copper Materials for Application as Reference Materials for High Precision Copper Isotope Analysis by Laser Ablation Inductively Coupled Plasma Multi-Collector Mass Spectrometry

**DOI:** 10.3389/fchem.2021.617205

**Published:** 2021-04-15

**Authors:** Zhaoping Yang, Simon Edward Jackson, Thomas Skulski

**Affiliations:** Geological Survey of Canada (GSC), Natural Resources Canada, Ottawa, ON, Canada

**Keywords:** copper isotopes, reference materials, LA-MC-ICP-MS, homogeneity, native copper

## Abstract

Laser ablation inductively coupled plasma multi-collector mass spectrometry (LA-MC-ICP-MS) allows rapid, *in situ,* highly precise measurements of Cu isotope ratios of native Cu and Cu-bearing minerals. However, the National Institute of Standards and Technology Cu-metal isotope standard NIST SRM976 that is commonly used to calibrate LA-MC-ICP-MS Cu isotope measurements of native Cu is no longer available. We have investigated the suitability of four Cu metal materials, SSC-1, SSC-3 and SSC-4 (cathode Cu metal rods) and CUPD-1 (Cu anode sawings), originally developed by the Canada Centre for Mineral and Energy Technology (CANMET) as certified reference materials for trace element analysis, as Cu isotope reference materials for LA-MC-ICP-MS analysis and solution nebulization (SN) of Cu. The Cu isotopic composition and homogeneity of these four materials were characterised by SN- and LA-MC-ICP-MS, and are reported for the first time. The bulk Cu isotopic compositions, expressed as δ^65^Cu_SRM976_ in per mil (‰) relative to NIST SRM976 with combined uncertainties (*U*, *k* = 2), of SSC-1, SSC-3 and SSC-4, determined utilizing SN-MC-ICP-MS, are identical within analytical uncertainty at 0.03 ± 0.07‰ (*n* = 29), 0.04 ± 0.04‰ (*n* = 28), and 0.05 ± 0.08‰ (*n* = 29), respectively; the composition of CUPD-1 is 2.14 ± 0.08‰ (*n* = 28). The compositions are 0.01 ± 0.07‰ (*n* = 29), 0.04 ± 0.06‰ (*n* = 29), 0.03 ± 0.06‰ (*n* = 28) and 2.15 ± 0.06‰ (*n* = 28), respectively, relative to the European Reference Material ERM®-AE633 Cu isotope standard. The Cu isotope homogeneity of the four new reference materials was assessed by determining whether multiple individual *in situ* Cu isotope measurements made by LA-MC-ICP-MS analysis (43 µm spot size), using each of the other three reference materials as a calibrator, approximate a single normal distribution. We also investigate whether there are statistically significant differences between the mean δ^65^Cu values of three independent data sets for each of the Cu isotope reference materials using one-way analysis of variance (ANOVA). Normality tests (graphical assessment of normal distribution quantile-quantile plots, and the Shapiro-Wilk, Jarque-Bera and reduced chi-squared statistic tests) show that: 1) the Cu isotope data acquired on SSC-1, SSC-3, SSC-4 and CUPD-1 do not depart significantly from a normal distribution, 2) the scatter of the Cu isotope data is due to analytical uncertainty with 95% confidence, and 3) there are no other significant sources of scatter; e.g. heterogeneity of the reference materials. The results of one-way ANOVA reveal that the mean difference of the δ^65^Cu value for each of the reference materials SSC-1, SSC-3, SSC-4 and CUPD-1 is statistically not significant at the 0.05 level. The mean δ^65^Cu_SRM976_ values with combined uncertainties (*U*, *k* = 2) of SSC-1, SSC-3, SSC-4 and CUPD-1, determined by LA-MC-ICP-MS using each of the other three reference materials as a calibration standard, are 0.03 ± 0.09‰ (*n* = 132), 0.05 ± 0.09‰ (*n* = 154), 0.03 ± 0.09‰ (*n* = 144) and 2.14 ± 0.10‰ (*n* = 106), respectively. These values are in agreement with those determined by SN-MC-ICP-MS analysis at the 95% confidence level and have excellent precision (2 s.d. ≤ 0.10‰). These results suggest that SSC-1, SSC-3, SSC-4 and CUPD-1 can be considered isotopically homogeneous at a spatial resolution of 43 μm, and they are suitable reference materials for calibration and quality control of *in situ* and solution nebulization Cu isotope analyses of Cu.

## Introduction

Copper is economically an extremely important metal in our industrial and technological society. Copper is commonly concentrated in ores in the form of copper sulphides, such as chalcocite (Cu_2_S), covellite (CuS), chalcopyrite (CuFeS_2_) and bornite (Cu_5_FeS_4_), and other ore minerals formed during hypogene and supergene ore mineralization processes (Faure 1991; Larson et al., 2003; Mathur et al., 2009a; Baxton and Mathur, 2011; Mathur et al., 2013; Mathur et al., 2018). However, more rarely, it can also occur as native Cu metal, which was highly prized for production of tools and weapons prior to development of smelting technologies.

Copper is siderophile and highly chalcophile (Siebert et al., 2011). It is also a redox-sensitive element, having two oxidation states - cuprous Cu (I) and cupric Cu (II) that, in addition to metallic Cu, occur in natural environments (Moynier et al., 2017). Copper has two stable isotopes, ^65^Cu and ^63^Cu, with a nominal relative mass difference of 3.2%. Copper’s multiple oxidation states and the significant relative mass difference of its isotopes result in measurable isotopic fractionation during chemical processes involving Cu during ore formation, with recorded δ^65^Cu values (^65^Cu/^63^Cu isotope amount ratio relative to a Cu isotope standard NIST SRM976 in per mil) ranging from −16.49‰ to +19.73‰ (Yuan et al., 2017).

The pioneering search for natural variations in Cu isotope ratios (Walker et al., 1958; Shields et al., 1965) was made using thermal ionization mass spectrometry (TIMS). Both of these investigations demonstrated Cu isotopic variations of several per mil in copper ores despite achievable analytical uncertainty poorer than 1.5‰ due to the time-dependent mass bias for Cu isotopes that was difficult to evaluate properly (Ikehata et al., 2008; Moynier et al., 2017). With the benefit of the much increased ionization efficiency of an ICP ion source, the advent of multi-collector inductively coupled plasma mass spectrometry (MC-ICP-MS) allowed precise and accurate measurement of Cu isotope ratios with analytical precision (2 s.d.) better than 0.08‰ (Gale et al., 1999; Maréchal et al., 1999; Zhu et al., 2000; Maréchal and Albarede, 2002; Archer and Vance, 2004; Mason et al., 2004a; Mason et al., 2004b). This made it possible to investigate the natural mass-dependent Cu isotope variations within single deposits where Cu isotopic variations are commonly < 2.0‰. In the 2 decades following the development of MC-ICP-MS, Cu isotope compositions, determined using solution nebulization (SN) MC-ICP-MS, have been used in studies of a wide spectrum of Cu ore-forming systems, including native Cu deposits (Zhu et al., 2000; Larson et al., 2003; Ikehata and Hirata, 2012; Dekov et al., 2013; Ikehata et al., 2015; Bornhorst and Mathur, 2017; Baggio et al., 2018) and also, increasingly, in archaeology for the purpose of investigating the provenance of Cu artefacts (Mathur et al., 2009b; Mathur et al., 2014; Wilson et al., 2016).

Coupling laser ablation (LA) sample introduction with MC-ICP-MS detection provided a new technique for rapid and precise *in situ* determination of Cu isotope ratios of native Cu and Cu-bearing minerals (Jackson et al., 2001; Jackson and Günther, 2003). The LA-MC-ICP-MS is especially important for resolving Cu isotope differences in Cu sulphides on fine scales (10’s of µm) (Ikehata et al., 2008; Ikehata and Hirata, 2013) where sampling extremely small domains by micro-drilling techniques for SN-ICP-MS analysis are almost impossible. Investigations of Cu isotopes, employing lasers with nanosecond (ns) pulse widths (Graham et al., 2004; Li et al., 2010) and lasers operating in the femtosecond (fs) regime (Ikehata et al., 2011; Ikehata and Hirata, 2013; Lazarov and Horn, 2015) have demonstrated that LA-MC-ICP-MS is a technique that can provide *in situ* measurements with precisions capable of resolving Cu isotope ratio variations in ore deposits. However, laser-induced isotopic fractionation, including transient and matrix-dependent isotopic fractionation effects (Jackson et al., 2001; Jackson and Günther, 2003; Hirata et al., 2003; Kuhn et al., 2007; Horn and von Blanckenburg, 2007; Poitrasson and d'Abzac, 2017) encountered in the determination of Cu isotope ratios by LA-MC-ICP-MS using ns lasers, pose a challenge for precise and accurate Cu isotope measurement. The source of this fractionation is generally considered to be related to an ablation mechanism that is dominated by thermal effects. This results in melting of the sample and generation of sample aerosols consisting of both condensed vapour and melt droplets, which are isotopically fractionated and which change in relative proportions with matrix and ablation time. Differential transport efficiencies of the different particle types, together with incomplete volatilisation of the larger particles (melt droplets) in the ICP (Jackson et al., 2001; Jackson and Günther, 2003; Horn and von Blanckenburg, 2007), generate the observed fractionation effects. Thus matrix-matched calibration standards and the standard-sample-standard bracketing (SSB) method are required to achieve accurate results and sufficient precision (ideally < 0.1‰) for resolving small natural isotopic variations present in individual primary native copper and copper sulphide minerals.

The Cu isotope standard NIST SRM976, a pure copper metal that has been used not only for solution nebulization but also for laser ablation Cu isotope analysis, is no longer available. New solution Cu isotope certified reference materials have been developed, including ERM®-AE633, ERM®-AE647 (Moeller et al., 2012), Cu isotope reference materials NWU-Cu-A and NWU-Cu-B (Yuan et al., 2017), and HICU-1 (Sullivan et al., 2020). These enable a continuous comparison of Cu isotope amount ratios in samples with NIST SRM976 for solution Cu isotope analyses. However, the lack of a suitable calibration standard is no doubt limiting widespread application of LA-MC-ICP-MS for *in situ* Cu isotope analysis of native Cu ores and artefacts.

In this contribution, we investigated the suitability of four Cu metal materials SSC-1, SSC-3, SSC-4 and CUPD-1 as Cu isotope reference materials for LA-MC-ICP-MS analysis. These materials were all certified by the Canada Centre for Mineral and Energy Technology (CANMET) as reference materials for trace element analysis of impurities in Cu. The Cu isotopic composition and homogeneity of these four new reference materials were characterised by SN- and LA-MC-ICP-MS, and are reported for the first time. The bulk Cu isotopic composition from dissolutions of 0.15–1.95 g size fragments of each of the reference materials was measured against NIST SRM976 and ERM®-AE633 Cu isotope standards by SN-MC-ICP-MS. Multiple individual *in situ* Cu isotope measurements were made by LA-MC-ICP-MS analysis of each of four new reference materials at 43 µm spot size, using each of other three reference materials as a calibration standard. These measurements allowed assessment of the Cu isotope homogeneity of the four new reference materials at the sampling scale using statistical approaches, including graphical assessment of normal distribution quantile-quantile plots, and the Shapiro-Wilk (Shapiro and Wilk, 1965, 1968; Royston, 1982), Jarque-Bera (Jarque and Bera, 1980, 1987) and reduced chi-squared statistic (mean square weighted deviation (MSWD); Wendt and Carl, 1991; Ludwig, 2003) tests, as well as one-way analysis of variance (ANOVA). Furthermore, the effects of laser induced isotopic fractionation of Cu during ns pulse LA-MC-ICP-MS isotopic analysis were investigated and the achievable precision and accuracy of *in-situ* Cu isotope measurements of native Cu were evaluated. Finally, the suitability of the Cu isotope reference materials was further validated by analysis of Cu isotope reference materials ERM®-AE647 and Romil Cu by SN-MC-ICP-MS, and a native copper sample, NMC 12864, by LA-MC-ICP-MS.

## Materials and Methods

### Standards and Samples

Two calibration standards were used for SN-MC-ICP-MS Cu isotope analysis. They were a solution Cu isotope standard NIST SRM976 prepared by digestion of a 0.4 g aliquot of the Cu metal standard at Queens University, Canada, and Institute for Reference Materials and Measurements (IRMM) ERM®-AE633 solution Cu isotope reference material. NIST SRM976 is certified for its absolute isotopic abundance ratio (^63^Cu/^65^Cu = 2.2440 ± 0.0021; Shields et al., 1964; Shields et al., 1965; Thomas, 1994). ERM®-AE633 was reportedly prepared directly from NIST SRM976 by the IRMM and has a nominally identical Cu isotopic composition (δ^65^Cu value of NIST SRM976 Cu relative to ERM®-AE633 = −0.01 ± 0.05‰) (Moeller et al., 2012).

The solution Zn isotope standard NIST SRM683, originally developed as a certified reference material for trace element analysis (Rasberry, 1988) and newly developed as a Zn isotopic reference material (Yang L. et al., 2018), was used for instrumental mass bias correction. Its isotope composition relative to JMC-Lyon, the Zn isotope reference material against which Zn isotope data are commonly reported (Maréchal et al., 1999), is δ^66^Zn = 0.12 ± 0.04‰ (Yang Y. et al., 2018). The Zn isotope reference material JMC‐Lyon, obtained from Johnson Matthey^©^, London United Kingdom, is nearly exhausted and no longer produced. Its δ^66^Zn = −0.29 ± 0.05‰ relative to IRMM-3702 (Moeller et al., 2012), where the IRMM-3702 is an available Zn isotope reference material from IRMM and is certified for absolute isotopic abundance ratios. The certified Zn isotope composition of IRMM-3702 is ^66^Zn/^64^Zn = 0.56397 ± 30 (Ponzevera et al., 2006). Thus, the calculated ^66^Zn/^64^Zn ratio of NIST SRM683 used in this study is 0.56388. Additionally, the Cu isotope reference material ERM®-AE647 Cu from IRMM and a commercial single-element Cu solution, Romil Cu (Romil Ltd., Cambridge, United Kingdom), that has been widely characterised for its Cu isotopic composition (Zhu et al., 2000; Chapman et al., 2006; Peel et al., 2008; Larner et al., 2011; Moeller et al., 2012) were used for quality control of the SN-MC-ICP-MS Cu isotope analyses. The δ^65^Cu values of ERM®-AE647 Cu and Romil Cu relative to NIST SRM976 Cu are 0.21 ± 0.04 and 0.18 ± 0.06‰, respectively (Moeller et al., 2012).

The four Cu metal materials being investigated as Cu isotope reference materials for LA-MC-ICP-MS analysis were all originally developed as certified reference materials for trace element analysis of impurities in Cu by the CANMET. These materials are no longer commercially available but significant quantities still exist for distribution^1^. SSC-1, SSC-3, and SSC-4 are commercial purity Cu metal rods. Weighed quantities of several elements were added to high-purity Cu anode swarf. The mixture was cold-pressed into pellets that were added to melted cleaned cathode Cu. The melt was poured into a mould and subsequently hot rolled into 8 mm diameter copper rods (Malson, 1975). Trace element contents range from 40 to 90 μg g^−1^ for Sn and Pb in SSC-1, Fe and Ni in SSC-3, and S and Fe in SSC-4; other trace elements that occur at significant levels (10–35 μg g^−1^) are S, Fe, Ni, and Zn in SSC-1, S, Zn, Ag, and Sn in SSC-3, and Ni, Zn, Ag, Cd, Sn, Sb, and Pb in SSC-4 (Malson, 1975).

CUPD-1 consists of sawings (60 μm to hundreds μm in diameter) of Cu anode (Salley and Leaver, 2009). It contains impurities of several trace elements, including, most importantly, Ni, As, Se, Ag and Sb in the range of 150–310 μg g^−1^, and Fe, Te, Pb, and Bi with contents in the range of 40–69 μg g^−1^ (Salley and Leaver, 2009); all other elements documented in the four Cu isotope reference materials occur at <10 μg g^−1^ (Malson, 1975; Malson, 2003; Cooper et al., 2008; Salley and Leaver, 2009). In addition, a native copper sample, NMC 12864, originating from the Coppermine River area, Northwest Territories, Canada, and sampled from the National Mineral Collection (NMC), Geological Survey of Canada, was also used for this study. The impurities in this sample include Hg (0.4–4.9 μg g^−1^), Zn (3–8 μg g^−1^), Ni (7–21 μg g^−1^), As (12–45 μg g^−1^), Ag (146–333 μg g^−1^) and Sn (9–163 μg g^−1^), and low trace amounts (0.01–1.25 μg g^−1^) of Co, Se, Sb, and Au (Cooper et al., 2008).

An aliquot of SSC-1, SSC-3, and SSC-4 (1.62–1.95 g) were cut from the Cu rods and polished to a 0.25 μm finish to remove the sawn surfaces. The CUPD-1 sawings (0.15 g) were checked and handpicked under a binocular microscope. These reference materials were rinsed three times with Milli-Q water and ethyl alcohol (C_2_H_5_OH) in turn, followed by three 15 min rounds of ultrasonic cleaning in each of ethyl alcohol and Milli-Q water. The materials were then rinsed three more times with Milli-Q water, and dried in a fume hood. Each of the reference materials was carefully weighed and placed in a 50 ml polytetrafluoroethylene (PTFE) beaker. The reference materials were digested using 9 ml of 8 M HNO_3_ followed by 3.1 ml of 16 M HNO_3_. The solutions were reduced to dryness on a hot plate at 100°C and then re-dissolved to produce 1,000–4,000 μg g^−1^ stock solutions in 0.4 M HNO_3_. All reagents used in this study were prepared using Optima™ high purity HNO_3_ from Fisher Scientific and >18 MΩ⋅cm deionized Milli-Q water.

For laser ablation analysis, two 25 mm round epoxy mounts were prepared, each containing an aliquot of each of the four reference materials. Another mount was prepared containing sample NMC 12864. The mounts were polished to a 0.25 μm finish. The sample mounts were then rinsed with Milli-Q water and cleaned in an ultrasonic bath for 15 min in Milli-Q water and ethyl alcohol successively. The surface of the mount was cleaned using ethyl alcohol immediately before LA-MC-ICP-MS Cu isotope analysis.

### Instrumentation and Data Acquisition

Copper isotope ratio measurements were performed using SN- and LA-MC-ICP-MS at the Geological Survey of Canada (GSC), Natural Resources Canada, in Ottawa, using a Thermo Scientific™ Neptune Plus™ High Resolution MC-ICP-MS. The SN Cu isotope measurements were performed in “wet plasma” mode. For laser ablation analysis, the MC-ICP-MS was coupled to a Teledyne Photon Machines Analyte G1 excimer laser ablation system (*λ* = 193 nm). Complete SN- and LA-MC-ICP-MS operating conditions are provided in Table 1. An electronic baseline measurement of 120 s and a Faraday collector gain calibration were performed daily before Cu isotope measurements started. The standard-sample-standard bracketing (SSB) external standardisation technique, in conjunction with Zn internal normalization (Maréchal et al., 1999; Maréchal and Albarède, 2002; Graham et al., 2004; Mason et al., 2004a; Maher, 2005; Yang L. et al., 2018), were used to correct for instrumental mass bias for both SN and LA analyses.

**TABLE 1:** SN- and LA-MC-ICP-MS operating conditions.

Laser Ablation system		
Model	Analyte G1 (Teledyne Photon Machines Inc., United States)
Sample cell	HelEx 2-volume cell
Wavelength	193 nm
Pulse duration(FWHM)	4 nm
Energy density	10.0 J cm^−2^
Repetition rate	2 Hz
Ablation mode	Spot analysis
Spot diameter	43 μm
Gas flows:	
Carrier He gas flow MFC-1 (cell cup)	0.45 L min^−1^
Carrier He gas flow MFC-2 (cell base)	0.70 L min^−1^
Make up Ar gas flow	0.85−0.90 L min^−1^
Signal smoothing	50 mL aerosol mixing tube
Particle filtering	Plug of glass wool (∼ 40 mg)
Pre-ablation	No

MC-ICP-MS		
Model	Neptune Plus High Resolution MC-ICP-MS (Thermo Scientific™, Germany)	
Cones	Standard H-type Ni cones	
Spray chamber	Glass type double pass Scott design	
Cup configuration	^62^Ni (L3), ^63^Cu (L2), ^64^Zn (L1), ^65^Cu (C), ^66^Zn (H1), ^67^Zn (H2), ^68^Zn (H3)	
Detectors	Faraday cups equipped with 10^11^ Ohm resistors	
Resolution	Low	
	Solution Analysis	Laser Analysis
Integration time	4.174 s	0.527 s
Cycles/block	20 (blank) and 40 (sample)	40 (blank) and 265 (sample)
Blocks	1	1
Background signal	84 s	40 s
Sample signal	167 s	120 s
Wash time	180 s	90 s

Aridus desolvating nebulization system		
Model	Aridus II™ (CETAC, Omaha, United States)	
Spray chamber temp	70 °C	
Desolvator temp	160 °C	
Ar sweep gas flow	5.11–6.33 L min^−1^	
N2 addition gas flow	3 mL min^−1^	
Nebulizer	PFA micro-flow	
Sample uptake	100 μL min^−1^	
NIST SRM683 solution Zn concentration	30 ng g^−1^	

For SN-MC-ICP-MS Cu isotope analysis, the bracketing calibrator and sample solutions were diluted to 100 ng g^−1^ Cu in 2% HNO_3_ and doped with Zn (NIST SRM683) to yield analysis solutions with 1:1 Cu:Zn concentration ratio. This ensures similar Cu and Zn ion signal intensities, and minimizes differences in isotope fractionation behaviour, which appears to be caused by space-charge effects in the interface region (Archer and Vance, 2004), and results in reduced errors in the mass bias estimation (Zhu et al., 2000; Archer and Vance, 2004). Each Cu isotope measurement consisted of 20 cycles and 40 cycles of 4.174 s integration time for the analyses of acid blank (2% HNO3) and Cu solutions, respectively. The analytical sequence was: blank, standard, blank, sample, blank, standard, blank, sample, etc. The instrument was flushed for 3 min using 2% HNO_3_ after each analysis. The total Cu signal intensity for the acid blank background was 4–6 mV for ^63^Cu and ^65^Cu and total Zn signal intensity was 4.0–5.0 mV for ^64^Zn and ^66^Zn. The total Cu signal intensity was 6.0–10 V for 100 ng g^−1^ Cu; the total Zn signal intensity was 2.7–3.5 V for 100 ng g^−1^ Zn in different analytical sessions. Each of the SSC-1, SSC-3, SSC-4, and CUPD-1 reference materials, along with the quality control Cu isotope reference material ERM®-AE647, were measured repeatedly in multiple analytical sessions using either NIST SRM976 or ERM®-AE633 as the Cu isotope calibration standard. The Cu isotope reference materials ERM®-AE647 and Romil Cu were also measured in different analytical sessions using SSC-1, SSC-3, SSC-4 or CUPD-1 as the Cu isotope calibration standard. Analyses with values outside of the mean ± 2 s.d. were repeated. The solution analyses were conducted over a period of a month.

Laser ablation sampling for MC-ICP-MS Cu isotope analysis was performed in a He atmosphere, which reduces dramatically the condensation blanket of material around the ablation site (Guillong and Günther, 2002) and improves the transport efficiency. All analyses were accomplished using 43 µm spots, a low laser repetition rate (2 Hz) and high laser fluence (10 J cm^−2^) to minimise down-hole laser-induced isotopic fractionation (Jackson and Günther, 2003). The ablation aerosol was first passed through a 50 ml volume test tube to smooth the signal. The aerosol was then passed through approximately 40 mg of glass wool (“glass wool superfine,” No. 41408002, Hecht-Assistent, Sondheim/Rhön, Germany) that was inserted into the 3.2 mm i. d. sample delivery tubing, creating an approximately 1.5 cm long plug. The glass wool filter preferentially filters larger (> 0.5 μm) aerosol particles that are too large to be quantitatively volatilised in the ICP and can result in noisy signals and severe isotopic fractionation (Jackson and Günther, 2003). The glass wool filter was replaced regularly (∼ 5 h for a maximum of 76 spot analyses) in order to minimise any cross contamination of the aerosol from particulates of completed analyses. A “semi-dry” aerosol of NIST SRM683 Zn, generated by an Aridus II™ desolvating nebulization system (CETAC, Omaha, United States), was added to the filtered ablation aerosol via a Y-shape connector placed between the glass wool filter and the ICP torch. The instrument was flushed for 90 s after each analysis. The total signal intensity for the gas background during LA-MC-ICP-MS analysis was ca. 3–5 mV for ^63^Cu and ^65^Cu. The total Zn background signal intensity was ca. 2–6 mV for ^64^Zn and ^66^Zn, respectively. The total Zn signal for 30 ng g^−1^ Zn was 4.6–5.4 V. The total Cu signal during ablation typically dropped from ca. 18–3.0 V during 120 s ablation time, for a mean of ca. 12 V. Each of SSC-1, SSC-3, SSC-4, and CUPD-1 was measured repeatedly using each of the other three materials, in turn, as a bracketing calibration standard. Analyses with values outside of the mean ± 2 s.d. were repeated. Laser ablation analyses were conducted over a period of two months.

### Data Reduction

Processing of the SN- and LA-MC-ICP-MS Cu isotope analysis data was performed in MS Excel spreadsheets. The time-resolved laser ablation signals were examined and signal intervals for integration were carefully selected. In most cases, the absolute variation of ^65^Cu/^63^Cu ratios for the individual measurement cycles was < 0.0005 and almost all of the acquired data (ca. 115 s of 120 s) were integrated. The raw Zn isotope signals, together with the calculated ^66^Zn/^64^Zn ratios, were also examined to ensure that there were no anomalous variations that might indicate localised differences in Zn contribution from extraneous sources, such as the samples being tested. The effect of the interference of ^64^Ni on ^64^Zn was also evaluated. The acid blank (2% HNO_3_)-corrected ^62^Ni^+^ signals for SN-MC-ICP-MS analyses were < 0.09 mV; thus ^64^Ni/^64^Zn signal intensity ratios were < 0.0014%. The gas blank-corrected ^62^Ni^+^ signals for LA-MC-ICP-MS were < 0.17 mV; thus ^64^Ni/^64^Zn signal intensity ratios were < 0.0022%. With ^64^Zn^+^ signals 45,000 times higher than ^64^Ni^+^ signal, the interference of ^64^Ni^+^ on ^64^Zn^+^ was deemed insignificant.

The acid or gas blank-corrected signal intensities for ^63^Cu^+^ and ^65^Cu^+^, along with signal intensities for ^64^Zn^+^ and ^66^Zn^+^, were used to determine the ^65^Cu/^63^Cu and ^66^Zn/^64^Zn isotope amount ratios for each acquisition cycle for the sample and bracketing calibrators. Then, the instrument mass bias factor (*f* ^Zn^) was calculated for both unknown samples and bracketing calibrators, using the exponential mass bias law, a ^66^Zn/^64^Zn isotope amount ratio of 0.56388 for NIST SRM683 and atomic mass data published by IUPAC (http://www.ciaaw.org/atomic-masses.htm, 2018). The determined *f* ^Zn^ was then applied to exponentially correct the measured ^65^Cu/^63^Cu isotope amount ratios for each analytical cycle of respective samples and bracketing calibrators.

In order to develop the most robust data processing procedure for LA-MC-ICP-MS Cu isotope analysis, the effect of removing individual outliers was evaluated by processing the data with and without outlier removal. Outliers were defined as a ^65^Cu/^63^Cu amount ratio for an individual cycle of a single analysis that deviated from the range of mean ± 2 sigma. The results from 707 analyses of SSC-1, SSC-3, SSC-4, and CUPD-1 show that an absolute difference between measured ^65^Cu/^63^Cu isotope amount ratios with and without outlier removal are 0.000003 for 80.2% analyses, 0.000008 for 15% analyses and 0.000016 for 3.4% analyses respectively. This indicates that measured ^65^Cu/^63^Cu isotope amount ratios obtained with outlier removal are not significantly different from those without outlier removal in most instances. In contrast to the results described above, only 1.4% of total analyses display significant differences (ranging from 0.000029 to 0.0030 absolute difference) between outlier-filtered and unfiltered Cu isotope amount ratios. Inspection of these significant outliers reveals that they are all clearly related to spikes in ^65^Cu/^63^Cu that appeared randomly in analyses and affected one cycle to three cycles of data. These spikes, which are commonly observed in LA analyses, were most likely induced by large ablated particles, not other instrument noise. Such spikes are probably related to release from the glass wool of large particles that are incompletely vaporized in the ICP-MS, resulting in ionization of fractionated Cu vapour.

This study demonstrates that for 98.6% of the analyses, the approach of outlier removal yields insignificant difference in the measured ^65^Cu/^63^Cu isotope amount ratios from the values for the same analyses not subjected to outlier removal. However, outlier analysis is considered critical for detecting and removing significant, erratic, analytical spikes during the analyses and thus ensuring a robust procedure for accurate and precise Cu isotope ratio measurements. Therefore, any outliers at the 2 sigma confidence level in the mass bias-corrected cycle values were discarded, both for SN and LA analyses. This resulted in filtering of 0 cycles to 4 cycles of data for SN analyses (40 cycles) and 2 cycles to 15 cycles for LA analyses (maximum 221 cycles).

Finally, the mean of the outlier-filtered, mass bias-corrected Cu isotope ratios of an unknown sample was further corrected by application of the SSB procedure to account for additional analytically-induced isotopic fractionation (e.g., laser-induced). The Cu mass bias factor (*f* ^Cu^) was determined using the exponential mass bias law and the true ^65^Cu/^63^Cu isotope amount ratio of the bracketing calibrator. The mean of the factors *f* ^Cu^ obtained for the two adjacent bracketing calibrators was then employed to achieve correction of additional isotopic fractionation and to calculate the mass bias-corrected ^65^Cu/^63^Cu amount ratio of an unknown sample. The determined absolute ^65^Cu/^63^Cu amount ratio of each unknown sample was then converted to standard delta notation (δ^65^Cu units), where δ^65^Cu is relative difference in per mil (‰) of the ^65^Cu/^63^Cu amount ratio of the sample and the NIST SRM976 standard (Shields et al., 1964; Shields et al., 1965) (Eq. 1), having a certified value of ^65^Cu/^63^Cu_SRM976_ = 1/2.2440 = 0.44563.

δ65Cu = (C65u/C63usampleC65u/C63ustandard −1)  (1)

The uncertainties, contributed from the mass correction factor, the true Cu isotope amount ratio in the bracketing calibrator, and normalization standard NIST SRM976, were estimated using the rule for propagation of errors. The combined uncertainty associated with the reported δ^65^Cu value was estimated according to JCGM 100, 2008 “GUM 1995 with minor corrections,” and in consideration of type A major uncertainty components associated with the measurement of δ^65^Cu value, including the standard uncertainties from an unknown sample and the calibration standard. Considering Eq. 1 and using the rules for propagation of uncertainty for uncorrelated input quantities, the square of combined uncertainty *U* ^2^ can be expressed as Eq. 2:

$$U^{2}\left(\delta^{65}\mathrm{Cu}\right)=\frac{1}{R_{standard}^{2}}\;\times U_{sample}^{2}+\frac{R_{sample}^{2}}{R_{standard}^{4}}\times U_{standard}^{2}\;$$ (2)

where, *U*_sample_ and *U*_standard_ are standard measurement uncertainty (standard deviation) of ^65^Cu/^63^Cu amount ratios of an unknown sample and the calibration standard, respectively, and *R*_sample_ and *R*_standard_ are mass bias corrected ^65^Cu/^63^Cu amount ratios of an unknown sample and the calibration standard, respectively. The expanded uncertainty can then be obtained by multiplying *U* by a coverage factor *k* = 2, which gives a confidence level of 95% (JCGM 100: 2008).

A graphical assessment of normal distribution by quantile-quantile plots [the quantiles of the sample data (ordered by δ^65^Cu values) vs. the theoretical quantile values for a normal distribution] and a Jarque-Bera statistic test for normality were completed in MS Excel spreadsheets. A Shapiro-Wilk test for normality and one-way analysis of variance (ANOVA) were conducted using the IBM SPSS Statistics software version 26. Calculation of the reduced chi-squared statistic, also known as mean square weighted deviation (MSWD), was performed using Isoplot 3.00: a Geochronological Toolkit for Microsoft Excel (Ludwig, 2003).

## Results and Discussion

### Effects of Matrix in SN-MC-ICP-MS

On the basis of the certified element concentrations in the Cu reference materials from previous studies (Malson, 1975; Malson, 2003; Cooper et al., 2008; Salley and Leaver, 2009), the determined concentrations of impurity trace elements such as S, Mn, Fe, Ni, Zn, Se, As, Ag, Cd, Sn, Sb, Te, Au, Pb, and Bi in the 100 ng g^−1^ Cu solutions of the reference materials were in the range of ≤ 0.031 ng g^−1^. The concentrations of other impurity elements in the same solutions determined in this study using a Thermo X-Series II quadrupole ICP-MS were < 6 ng g^−1^ for Na, Mg, Al and Ca, and < 0.5 ng g^−1^ Ti, V, Cr, and Ba. Therefore, the calculated concentration ratios of most impurity elements, relative to Cu, in the pure copper reference materials were in the range of < 0.0003; and in the range of < 0.1 for Mg, Al, S and Ca. These concentration ratios are in the range that are reported to produce insignificant matrix related biases according to studies on matrix effects in SN-MC-ICP-MS Cu isotope measurement. These include the effects of Fe (Zhu et al., 2002; Graham et al., 2004; Rouxel et al., 2004; Maher, 2005; Markl et al., 2006; Maher and Larson, 2007), S (Rouxel et al., 2004), Zn (Graham et al., 2004; Maher, 2005), Ni (Graham et al., 2004; Maher, 2005), Na (Zhu et al., 2002; Savage et al., 2015); Mg (Zhu et al., 2002). Furthermore, given the low levels of impurities and low production rate of polyatomic ion interferences in ICP-MS, potential polyatomic interferences from impurity elements in 100 ng g^−1^ Cu solutions (e.g., ^23^Na^40^Ar^+^, ^27^Al^36^Ar^+^, ^47^Ti^16^O^+^, ^126^Te^2+^ on ^63^Cu^+^; ^24^Mg^40^Ar^+^, ^28^Si^36^Ar^+^, ^32^S_2_^+^, ^48^Ca^16^O^+^, ^48^Ti^16^O^+^, ^128^Te^2+^ on ^64^Zn^+^; ^25^Mg^40^Ar^+^, ^27^Al^38^Ar^+^, ^29^Si^36^Ar^+^, ^32^S^33^S^+^, ^49^Ti^16^O^+^, ^130^Te^+^, and ^130^Ba^2+^ on ^65^Cu^+^; ^26^Mg^40^Ar^+^, ^30^Si^36^Ar^+^, ^32^S^34^S^+^, ^50^Ti^16^O^+^, ^50^Cr^16^O^+^, and ^132^Ba^2+^ on ^66^Zn^+^) are considered to have been negligible.

The effect of Zn present in SSC-1, SSC-3, SSC-4 and CUPD-1 (Malson, 1975; Malson, 2003; Cooper et al., 2008; Salley and Leaver, 2009) on the accuracy of Zn isotope ratio measurement of NIST SRM683 in Cu isotope analysis of these reference materials by SN-MC-ICP-MS was also assessed. There was ca. 0.0015–0.0033 ng g^−1^ Zn impurity in the 100 ng g^−1^ Cu solution of those isotope reference materials spiked with 100 ng g^−1^ Zn NIST SRM683. Applying isotopic mixture theory (Faure and Mensing, 2005), the Zn isotopic composition in 100 ng g^−1^ Cu and Zn solutions can be considered a mixture of two Zn isotope components, which have different ^66^Zn/^64^Zn isotope amount ratios and different Zn concentrations, including 100 ng g^−1^ NIST SRM683 Zn solution, and impurity Zn in 100 ng g^−1^ Cu solution of the Cu isotope reference materials. If it is assumed that the Zn isotope composition of the Zn impurity in each Cu isotope reference material has the most extreme value reported for terrestrial samples (δ^66^Zn_JMC-Lyon_ = 2.0‰; Moynier et al., 2017), the calculated ^66^Zn/^64^Zn isotope composition of the Zn impurity in the Cu isotope reference materials would be 0.564934. Thus, the ^66^Zn/^64^Zn isotope composition of the Zn isotope mixture solution, calculated using the equation of isotope ratio mixtures (Faure and Mensing, 2005), is nominally identical to the 0.563880 value of NIST SRM683 with an absolute relative difference < 0.000007%. Therefore, Zn impurities in the reference materials are expected to have produced no measurable effect on the measured ^66^Zn/^64^Zn isotope composition of NIST SRM683 in SN analyses and subsequently no significant bias in the mass bias correction factors for the Cu isotope measurements.

### Solution Nebulization MC-ICP-MS Measurements

**FIGURE 1:**
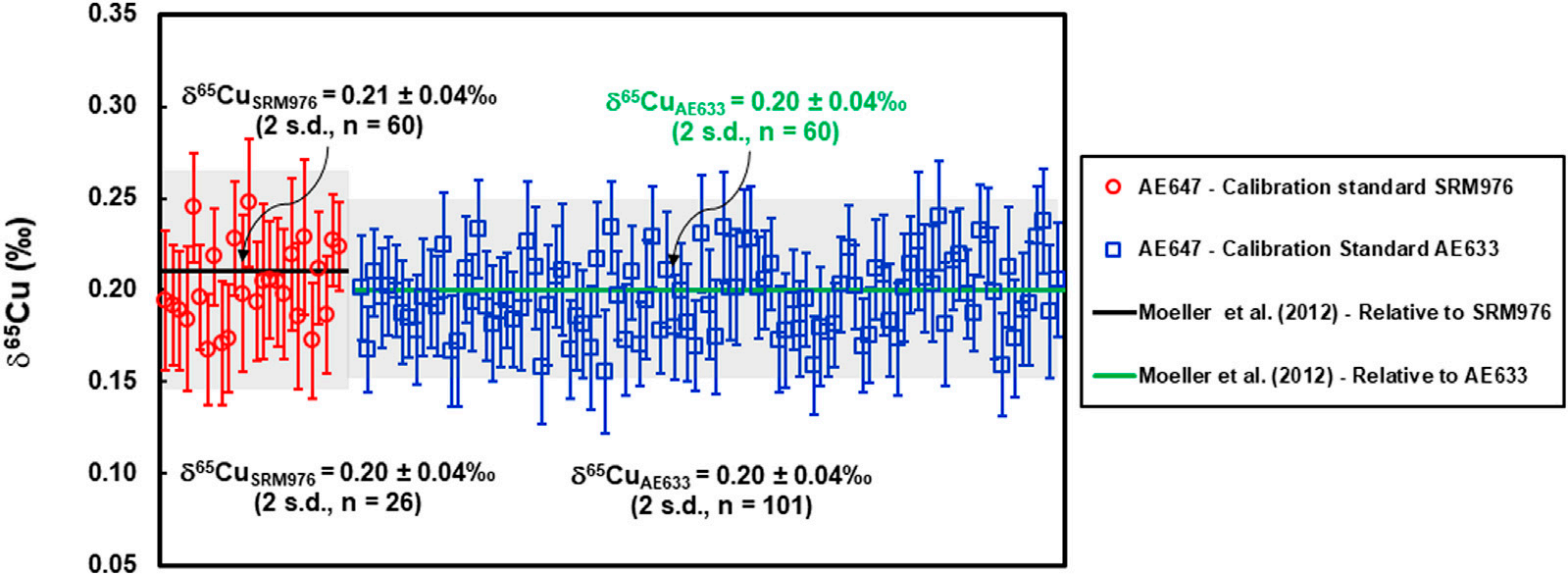
Cu isotope determinations of certified reference material ERM®-AE647. The determined δ^65^Cu values, measured against NIST SRM976, were consistent during one analytical session in February 2019, and agree within uncertainty with the certified δ^65^Cu_SRM976_ value and precision from Moeller et al. (2012) (as indicated by a solid black line and shaded area). The determined δ^65^Cu values against ERM®-AE633 were consistent over three analytical sessions, measured from September 2018 to February 2019 and also agree well within the uncertainty with the certified δ^65^Cu_AE633_ values and precision from Moeller et al. (2012) (as indicated by a solid green line and shaded area). Precision of a single measurement is shown at 2 standard errors (number of measurement cycles, *n* = 40).

The measured δ^65^Cu_SRM976_ value for Cu isotope standard solution ERM®-AE647, analysed for quality control by SN-MC-ICP-MS, was 0.20 ± 0.07‰ (*U*, *k* = 2, *n* = 26), whereas the measured δ^65^Cu_AE633_ value was 0.20 ± 0.06‰ (*U*, *k* = 2, *n* = 101). These results, shown in Figure 1; Table 2, are in excellent agreement with previously reported values of δ^65^Cu_SRM976_ = 0.21 ± 0.04‰ (2 s.d., *n* = 60) and δ^65^Cu_AE633_ = 0.20 ± 0.04‰ (2 s.d., *n* = 60) (Moeller et al., 2012).

**TABLE 2:** δ^65^Cu values of reference materials ERM®-AE647, SSC-1, SSC-3, SSC-4 and CUPD-1 determined by SN-MC-IC-MS using NIST SRM976 and ERM®-AE633 as calibration standards.

Reference Materials	δ^65^Cu_SRM976_ (‰)	*U* (‰, k = 2)^a^	2 s.d. (‰)^b^	n	δ^65^Cu_AE633_ (‰)	*U* (‰, k = 2)^a^	2 s.d. (‰)^b^	n	Sources of materials	Sources of data
SSC-1	0.03	0.07	0.04	29	0.01	0.07	0.05	29	CANMET	This study
SSC-3	0.04	0.04	0.03	28	0.04	0.06	0.04	29		
SSC-4	0.05	0.08	0.05	29	0.03	0.06	0.03	28		
CUPD-1	2.14	0.08	0.06	28	2.15	0.06	0.04	28		
ERM®-AE647	0.20	0.07	0.04	26	0.20	0.06	0.04	101	IRMM	
	0.21		0.04	60	0.20		0.04	60		Moeller et al. (2012)

^a^ Combined measurement uncertainty, coverage factor *k* = 2 produces an interval having a level of confidence of approximately 95%.

^b^ Precision is given as 2 standard deviation of the repeated measurements.

**FIGURE 2:**
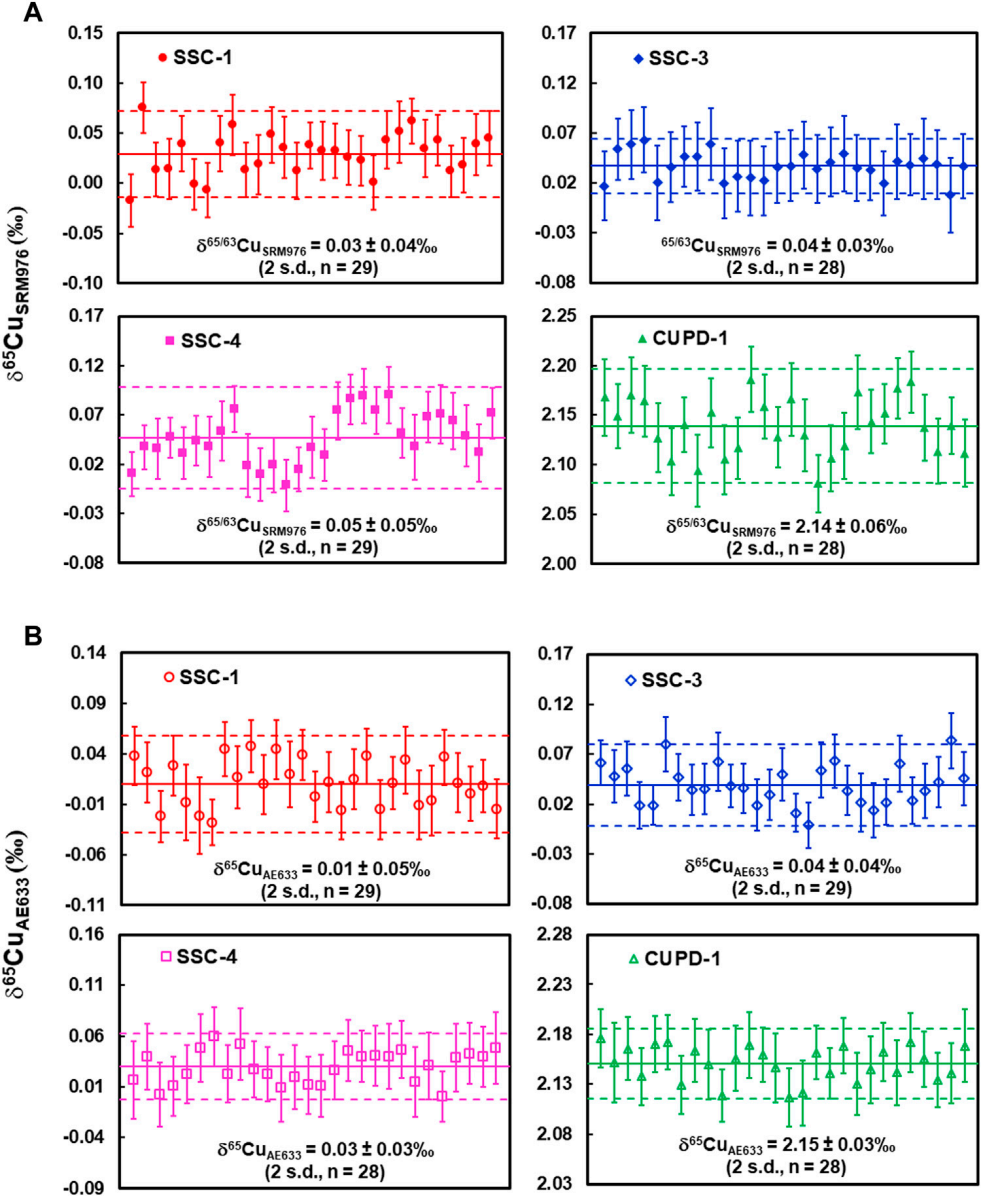
**(A)** SN-MC-ICP-MS Cu isotope determinations of reference material SSC-1, SSC-3, SSC-4 and CUPD-1 against NIST SRM976; **(B)** SN-MC-ICP-MS Cu isotope determinations of reference material SSC-1, SSC-3, SSC-4 and CUPD-1 against ERM®-AE633. Measurement precision is indicated by dashed lines. For a single measurement, the within-run precision was 0.03% (2 standard error; number of measurement cycles *n* = 40).

The measured δ^65^Cu_SRM976_ values of the SSC-1, SSC-3, SSC-4, and CUPD-1 solutions, determined with NIST976 as a calibration standard, were 0.03 ± 0.07‰ (*U*, *k* = 2, *n* = 29), 0.04 ± 0.04‰ (*U*, *k* = 2, *n* = 28), 0.05 ± 0.08‰ (*U*, *k* = 2, *n* = 29) and 2.14 ± 0.08‰ (*U*, *k* = 2, *n* = 28), respectively (Figure 2A; Table 2). The measured δ^65^Cu_AE633_ values of the same solutions, determined with solution ERM®-AE633 as a calibration standard, were 0.01 ± 0.07‰ (*U*, *k* = 2, *n* = 29), 0.04 ± 0.06‰ (*U*, *k* = 2, *n* = 29), 0.03 ± 0.06‰ (*U*, *k* = 2, *n* = 28) and 2.15 ± 0.06‰ (*U*, *k* = 2, *n* = 28), respectively (Figure 2B; Table 2).

**FIGURE 3:**
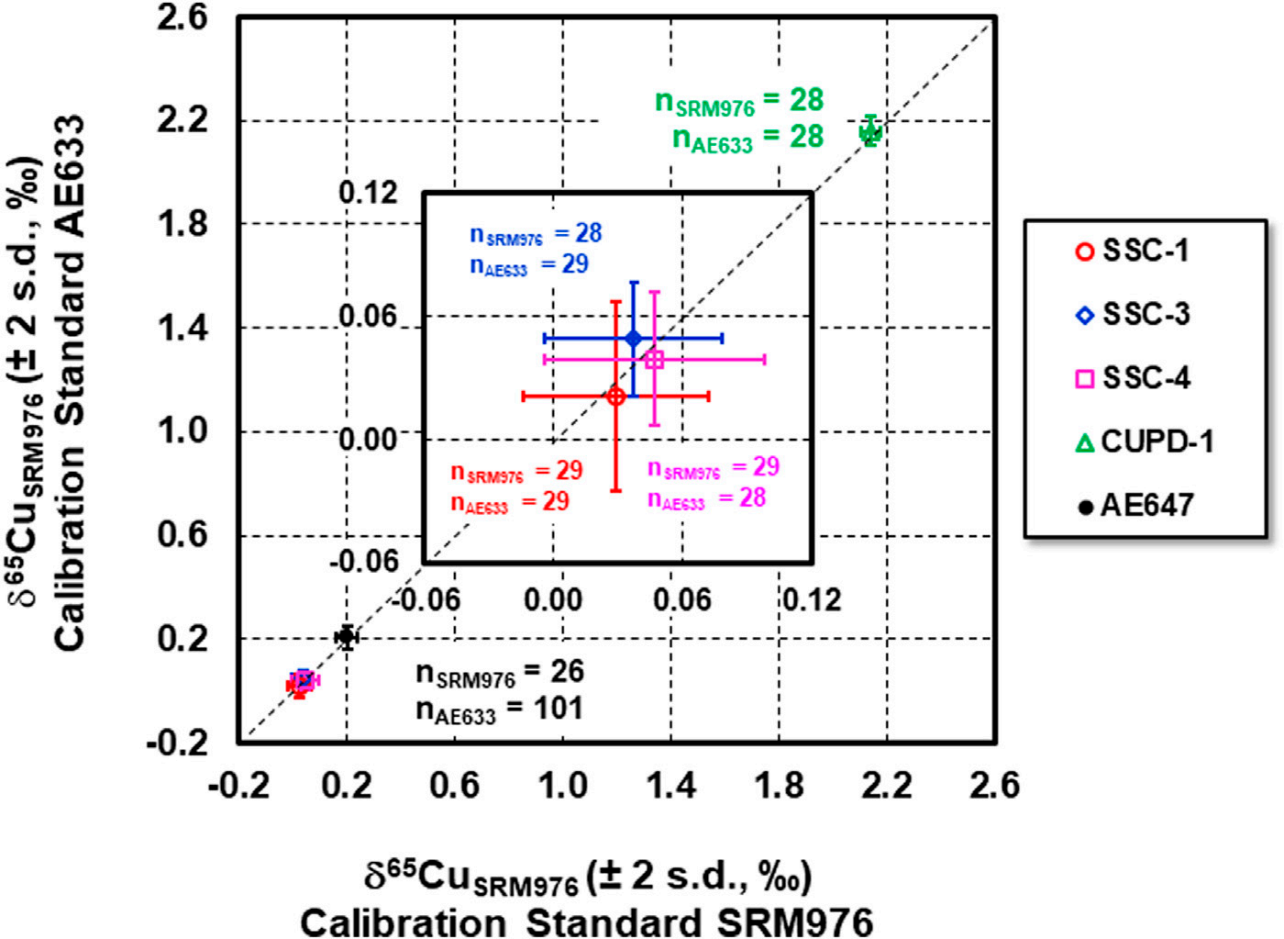
δ^65^Cu_SRM976_ values of reference materials SSC-1, SSC-3, SSC-4, CUPD-1 and ERM®-AE647 determined using calibration standard NIST SRM976 vs. using ERM®-AE633.

The measured δ^65^Cu_AE633_ values of the new Cu isotope reference materials and reference material ERM®-AE647, determined using ERM®-AE633 as a calibration standard, were recalculated as δ^65^Cu_SRM976_ values on the basis that the relative difference in δ^65^Cu of NIST SRM976 and ERM®-AE633 = −0.01 ± 0.05‰ (2 sd., *n* = 40) (Moeller et al., 2012). These values were then plotted against the δ^65^Cu_SRM976_ values for the same materials using NIST SRM976 as a calibration standard (Figure 3). The Cu isotope data overlap the 1:1 relationship within uncertainty, suggesting that measured relative Cu isotope amount ratios for each of the new Cu isotope reference materials and reference material ERM®-AE647, using ERM®-AE633 as a calibration standard are identical at the 95% confidence level to the ratios determined using NIST SRM976 as a calibration standard. Moreover, the δ^65^Cu values for Cu isotope standard solution ERM®-AE647 and Romil Cu, determined using each of the newly developed reference materials, SSC-1, SSC-3, SSC-4 and CUPD-1, as a calibration standard (Supplementary Figure S1A; Figure 1B; Supplementary Table S1), show that mean measured δ^65^Cu of ERM®-AE647 was 0.18 ± 0.06‰ (2 s.d., *n* = 69), which is in good agreement with the certified δ^65^Cu values 0.21 ± 0.04‰ (2 s.d., *n* = 60; Moeller et al., 2012). The mean measured δ^65^Cu value of Romil Cu was 0.18 ± 0.07‰ (2 s.d., *n* = 68), and is also in good agreement with the reported δ^65^Cu of 0.18 ± 0.06‰ (2 s.d., *n* = 19) and 0.21 ± 0.08‰ (2 s.d., *n* = 69) obtained in two different laboratories (Moeller et al., 2012).

For relative Cu isotope amount ratio measurements by SN-MC-ICP-MS, a long term measurement precision was ± 0.04‰ (2 standard deviation, *n* = 101 over 1 month), estimated based on analyses of the certified reference materials ERM®-AE647. For a single measurement, the precision was ± 0.03‰ (2 standard error; number of cycles = 40), estimated based on analyses of reference material ERM®-AE647 in this study. The long term and single measurement precision achieved in this study are comparable to the reported precisions of ± 0.05‰ (n = 60) and ± 0.05‰ (n = 40), respectively (Moeller et al., 2012).

### Effects of Laser-Induced Cu Isotopic Fractionation

**FIGURE 4:**
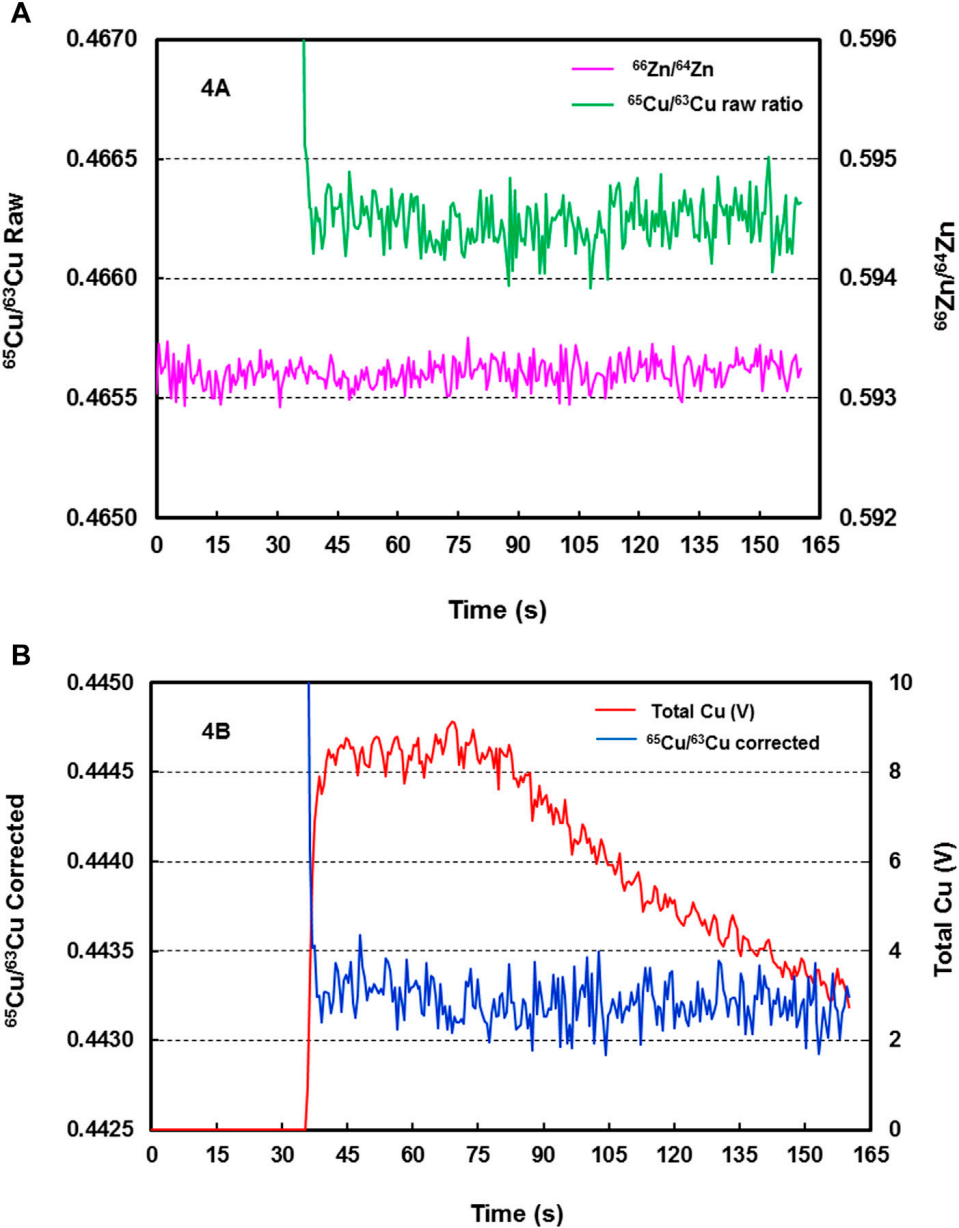
**(A)** Raw time-resolved ^65^Cu/^63^Cu isotope ratios (green line) and ^66^Zn/^64^Zn ratios (pink line) from LA-MC-ICP-MS analysis of reference material SSC-1 (DE06B22 analysis). Zn was added to the sample carrier gas as a dry aerosol. The relatively stable Cu isotope ratios indicate that substantial ablation time-dependent fractionation of Cu isotope ratios during the course of a ns laser ablation spot analysis was largely avoided by aerosol filtering. **(B)** Time resolved ^65^Cu/^63^Cu ratios corrected (blue line) for mass bias from LA-MC-ICP-MS analysis of reference material SSC-1 (DE06B22 analysis). The relatively stable Cu isotope ratios are entirely independent of the Cu signals (red line), and laser induced isotope fractionation was largely avoided by aerosol filtering.

During LA-MC-ICP-MS analysis, the raw and mass bias-corrected ^65^Cu/^63^Cu absolute isotope ratios were typically relatively stable during the 120 s data acquisition times (Figures 4A,B), whereas the ^63^Cu^+^ and ^65^Cu^+^ signals were typically stable for the first 35 s of ablation, following which signal strength decreased progressively during the subsequent 85 s (Figure 4B). These observations, which are consistent with the study of Jackson and Günther (2003), suggest that ablation time-dependent fractionation of Cu isotope ratios during the course of a ns laser ablation spot analysis was substantially avoided by aerosol filtering. The magnitude of Cu isotope variation during the whole data acquisition in the example shown in Figure 4 was 0.03‰.

It was also observed in our study that, under nominally identical laser ablation conditions, one analysis could display almost no ablation time-dependent fractionation of Cu isotope ratios during the whole period of ablation, whereas the next analysis could show obvious fractionation of Cu isotope ratios. These variations were consistently characterised by an increase of ∼ 0.08‰ in measured Cu isotope composition between the first and second 30 s of data. The cause(s) of this is not clear, but the consistent nature of the variations argues against isotopic heterogeneity of the sample. They are more likely associated with errors in laser focus position and/or fluctuations in laser output energy. Errors in the focus position would have resulted in a change of the pulse energy applied to the sample surface (Lazarov and Horn, 2015), which, combined with changes in laser output energy, could affect ns laser-copper interaction, and change the ablation efficiency due to the heat conduction losses and the plasma shielding effects (Semerok et al., 2002). It should be noted that even a within-run variation of 0.08‰ implies excellent performance when compared with Cu isotope variation of ca. 1.0‰ during a 90 s filtered ablation of pure copper using 213 ns LA analysis (Kuhn et al., 2007). Although this difference is not fully understood, it may be attributed to the entirely different LA and MC-ICP-MS hardware and settings employed in our study compared to those employed by Kuhn et al. (2007); for example, glass wool vs. centrifugal particle filtering, 2 vs. 5 Hz laser repetition rate and fluence of 10 vs. 17 J cm^−2^, respectively. The latter two settings presumably resulted in large differences in crater aspect ratios. It should also be noted that, even analyses showing within run variations of up to 0.08‰ provided measured Cu isotopic compositions that were indistinguishable within analytical uncertainty from the composition of the SN analysis of the same reference material.

The relative difference between internally (Zn) normalised SN Cu isotope ratio measurements of NIST SRM976 and the true value were 1.1–1.7% (0.62–0.95‰ absolute positive offset, *n* = 74). For LA analysis of SSC-1 and SSC-4, this difference, which represents the bias not corrected using Zn internal normalization alone, were 11.2–12.4% (5.3–5.7‰ negative offset, *n* = 37). The magnitude of the internal (Zn) mass bias correction for LA analyses (2.5–2.7%) was similar to that for SN analyses (2.6–2.8%), implying that the mass discrimination taking place in the ICP-MS as a result of plasma load and space charge effects, was very similar for both sample introduction systems. Thus, we estimate that laser sampling-induced Cu isotopic fractionation represents ∼ 10% (absolute) of the total analytically-induced isotopic fractionation budget. The large discrepancy between the mass bias-corrected Cu isotope ratios in LA analysis and the target Cu isotopic composition observed in this study is consistent with the results of Jackson and Günther (2003), and highlights the inability of Zn internal normalization to correct for laser-induced isotopic fractionation. On the other hand, this study shows that the achievable measurement precision (2 s.d.) attained in LA Cu isotope analysis using the SSB mass bias correction only, is 5 times to 7 times worse than the 0.07‰ achievable measurement precision obtained for the same analyses (*n* = 35) using both internal normalisation and SSB mass bias corrections. This indicates that there were significant instrumental drift effects during LA Cu isotope analysis that cannot be corrected by SSB alone. Thus, the approach of combined internal Zn normalisation and SSB bracketing is demonstrated to provide a highly effective and robust technique for precise and accurate Cu isotope ratio measurement by ns pulse LA-MC-ICP-MS.

### Homogeneity of the Reference Materials

Each of the reference materials was analyzed repeatedly using each of the three other reference materials as a calibrator. The Cu isotope homogeneity of the four new reference materials was assessed firstly by determining whether multiple individual *in situ* Cu isotope measurements made by LA-MC-ICP-MS analysis at 43 µm spot size approximated a single normal distribution. It was further assessed using one-way analysis of variance (ANOVA) to determine whether there are statistically significant differences between the mean δ^65^Cu values of three independent data sets for each of the Cu isotope reference materials, SSC-1, SSC-3, SSC-4, and CUPD-1. The results of the homogeneity tests of the reference materials are summarized in Tables 3, 4.

**TABLE 3:** Results of Cu isotope homogeneity tests for the reference materials SSC-1, SSC-3, SSC-4 and CUPD-1.

Reference Materials	δ^65^Cu_SRM976_ (‰)	2 s.d. (‰)	n	Normality Test^a^							
				MSWD^b^	Probability	Shapiro-Wilk^b^		Jarque-Bera^c^		Q-Q Plot
						Statistic W	Probability	Statistic χ^2^	Probability	*R* ^2^
Calibration standard SSC-1										
SSC-3	0.04	0.09	43	1.03	0.42	0.98	0.64	1.10	0.58	0.98
SSC-4	0.03	0.08	32	0.90	0.62	0.99	0.95	0.86	0.69	0.98
CUPD-1	2.14	0.09	46	1.04	0.40	0.97	0.22	1.98	0.37	0.97
Calibration standard SSC-3										
SSC-1	0.03	0.08	46	0.90	0.66	0.98	0.42	1.73	0.42	0.98
SSC-4	0.03	0.07	82	0.97	0.56	0.98	0.30	1.03	0.60	0.98
CUPD-1	2.13	0.09	38	1.30	0.13	0.98	0.54	1.21	0.55	0.98
Calibration standard SSC-4										
SSC-1	0.05	0.06	34	0.70	0.90	0.97	0.57	0.045	0.98	0.97
SSC-3	0.05	0.07	73	0.88	0.75	0.98	0.47	1.81	0.40	0.99
CUPD-1	2.13	0.08	22	0.99	0.47	0.96	0.55	1.23	0.54	0.97
Calibration standard CUPD-1										
SSC-1	0.03	0.08	52	0.80	0.87	0.98	0.70	1.03	0.60	0.99
SSC-3	0.04	0.07	38	0.83	0.76	0.99	0.92	0.49	0.78	0.99
SSC-4	0.04	0.09	30	1.12	0.30	0.95	0.19	1.70	0.43	0.96

^a^ Significance level in a normality test is α = 0.05.

^b^ Degrees of freedom are *n-1*.

^c^ Degrees of freedom are 2; **R*^2^*: correlation coefficient between the observed and expected normal values.

**TABLE 4:** Results of one-way ANOVA.

Reference Materials	Source	Sum of Square (SS)	Degrees of Freedom (DF)	Mean Square (MS)	F-statistic	Significance
SSC-1	Between Groups (SS_b_)	0.008	2	0.004	2.782	0.066
	Within Groups (SS_w_)	0.179	129	0.001		
	Total (SS_T_)	0.186	131			
SSC-3	Between Groups (SS_b_)	0.005	2	0.002	1.746	0.178
	Within Groups (SS_w_)	0.204	151	0.001		
	Total (SS_T_)	0.209	153			
SSC-4	Between Groups (SS_b_)	0.002	2	0.001	0.632	0.533
	Within Groups (SS_w_)	0.215	141	0.002		
	Total (SS_T_)	0.217	143			
CUPD-1	Between Groups (SS_b_)	0.008	2	0.004	2.260	0.110
	Within Groups (SS_w_)	0.186	103	0.002		
	Total (SS_T_)	0.194	105			

**FIGURE 5:**
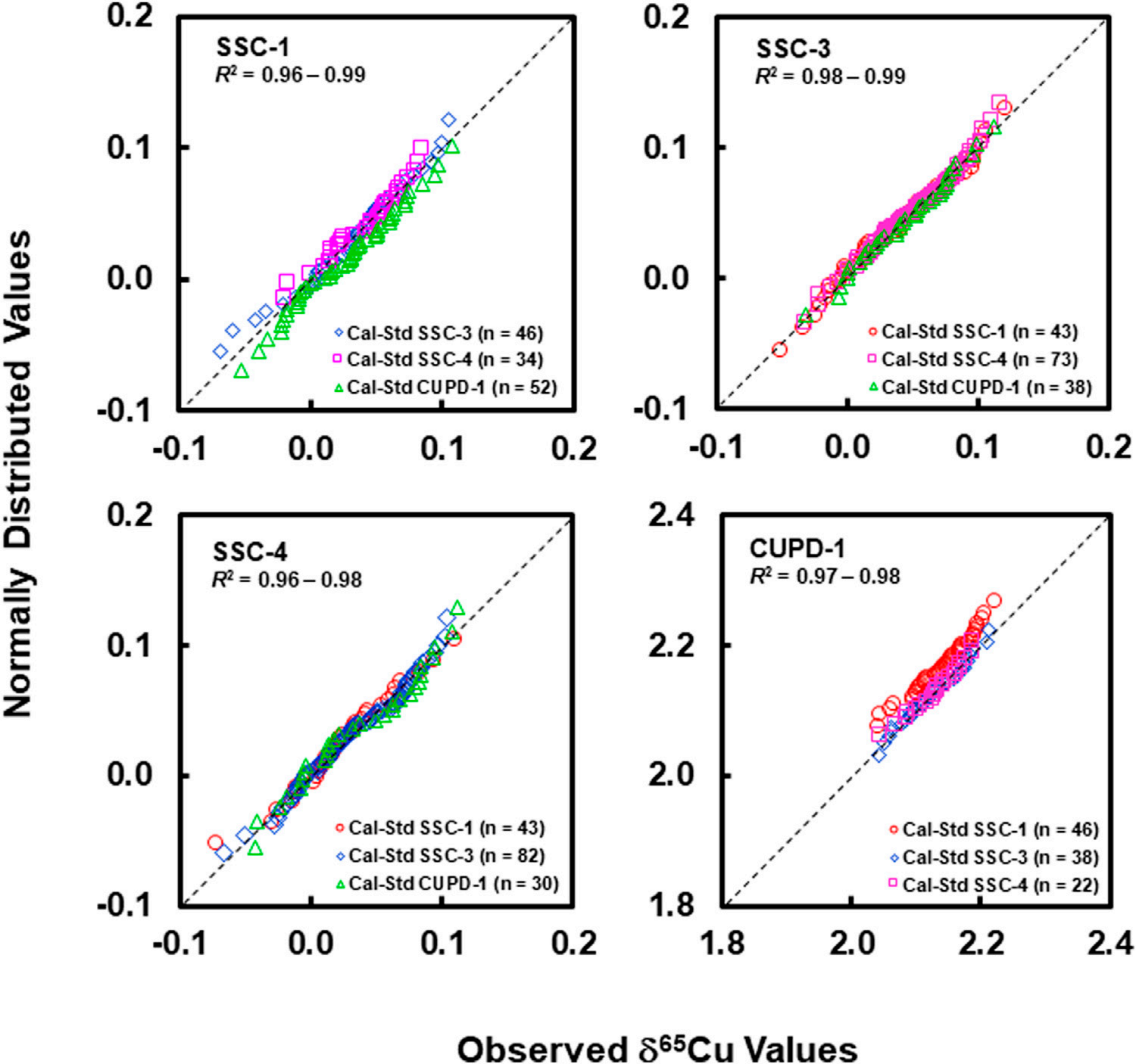
Normal quantile-quantile plots for SSC-1, SSC-3, SSC-4, and CUPD1 show that the quantile data points for the determined δ^65^Cu values for each of the reference materials do not seriously deviate from the 1:1 line and have linear correlation coefficients close to 1.

Graphical assessment of normal distribution using quantile-quantile plots (Q-Q) in Figure 5 shows that the quantile data points for the determined relative Cu isotope amount ratios from each of the reference materials did not deviate seriously from the fitted straight 1:1 line, and have good linear correlation coefficients close to 1 (0.96–0.99).

Normality tests using the well-known Shapiro-Wilk test (Shapiro and Wilk, 1965, Shapiro and Wilk, 1968; Royston, 1982) yielded statistic W values in the range of 0.97–0.98 (probability: 0.42–0.70) for SSC-1, 0.98 to 0.99 (probability: 0.47–0.92) for SSC-3, 0.95 to 0.99 (probability: 0.19–0.95) for SSC-4 and 0.96 to 0.97 (probability: 0.22–0.55) for CUPD-1. Statistic W values close to 1, with corresponding probability values significantly greater than 0.05, indicate that the Cu isotope data acquired on each of SSC-1, SSC-3, SSC-4 and CUPD-1 reference materials did not significantly depart from normality with 95% confidence.

An alternative test of normality, the Jarque-Bera test (Jarque and Bera, 1980; Jarque and Bera, 1987), a procedure for skewness-kurtosis testing, gave χ^2^ values in the range of 0.045–1.98 (probability: 0.37–0.78), which lies within the null hypothesis acceptance zone of less than the critical χ^2^ value of 5.448 (for *n* < 100) of the chi-squared distribution with 2 degrees of freedom at significance level *α* = 0.05. This reveals that Cu isotope data acquired on each of the new reference materials do not deviate significantly from a normal distribution with 95% confidence.

The homogeneity of the reference materials was also evaluated using the reduced chi-squared statistic, or MSWD (Wendt and Carl, 1991; Ludwig, 2003), at the 95% confidence interval, a measure of goodness of fit that takes into account the effect of both the internal and external analytical uncertainties. An MSWD value of approximately 1 indicates that the scatter of the data for a reference material can be explained by analytical uncertainties alone, and that additional sources of uncertainty, such as heterogeneity of the sample material, do not exist within measurement uncertainty (Wendt and Carl, 1991; Gilbert et al., 2014). The determined MSWD values were in the range of 0.70–0.90 (probability: 0.66–0.90) for SSC-1, 0.83–1.03 (probability: 0.42–0.76) for SSC-3, 0.90–1.12 (probability: 0.30–0.56) for SSC-4, and 0.99–1.30 (probability: 0.13–0.47) for CUPD-1, respectively. The MSWD values are all close to 1 and within the expected acceptance ranges of 1 ± 2 (2/*f*)^1/2^, where the degrees of freedom *f* = n–1 and n = the number of data points (Wendt and Carl, 1991), implying that the scatter of the Cu isotope data was due to the analytical uncertainty with 95% confidence, and no other significant sources of uncertainty were unaccounted for.

The homogeneity of the reference materials was further evaluated through one-way ANOVA to examine whether any of the three means of the relative Cu isotope ratios of a reference material measured using the other three reference materials as calibrators are different from each other with statistical significance. The results of one-way ANOVA (Table 4) illustrate that the mean difference for each of the reference materials SSC-1, SSC-3, SSC-4, and CUPD-1 is statistically not significant at the 0.05 level as determined with *F*_(2, 131)_ = 2.782, and *p* = 0.066 for SSC-1, *F*_(2, 153)_ = 1.746, and *p* = 0.178 for SSC-3, *F*_(2, 143)_ = 0.632, and *p* = 0.533 for SSC-4, and *F*_(2, 105)_ = 2.260 and *p* = 0.110 for CUPD-1.

The results of intensive homogeneity testing on two sections for each of the reference materials demonstrate that the new Cu isotope reference materials SSC-1, SSC-3, SSC-4 and CUPD-1 can be considered isotopically homogeneous at a spatial resolution of 43 µm with 95% confidence. It should also be noted that short ablations for some thin grains of CUPD-1, owing to rapid penetration of the laser through the grain, resulted in larger counting statistics-associated within-run measurement precision (2 standard error > 0.1‰). Therefore, LA analyses of CUPD-1 with less than 20 s laser ablation time were not considered in this study. Consequently, there are fewer Cu isotope data for CUPD-1 than for the other three reference materials. Further homogeneity tests should be conducted to unequivocally confirm isotopic homogeneity of CUPD-1.

### Laser Ablation MC-ICP-MS Measurement

**FIGURE 6:**
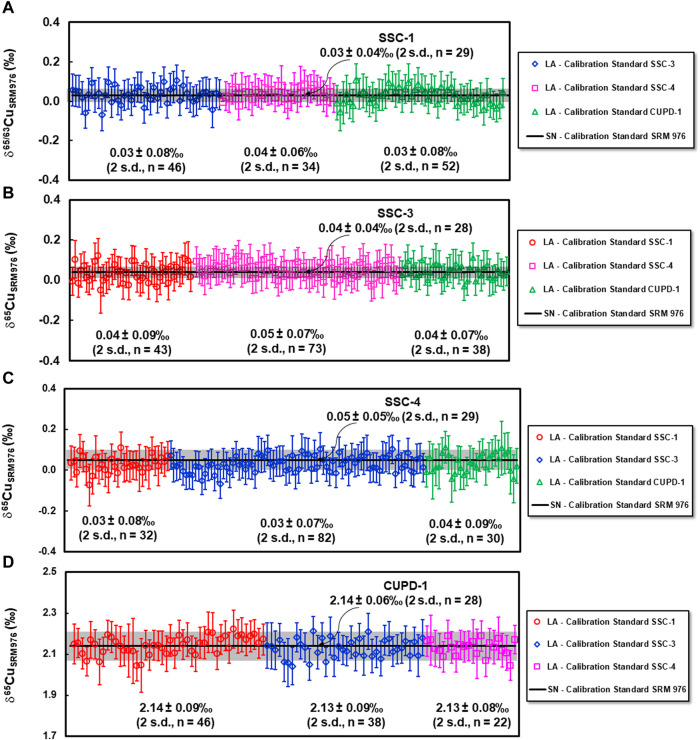
Results of Cu isotope ratio determinations of reference materials SSC-1 **(A)**, SSC-3 **(B)**, SSC-4 **(C)**, and CUPD-1 **(D)** by LA-MC-ICP-MS using each of the other three reference materials as a calibration standard. For a single measurement, the within-run precision is 2 standard error. For comparison, δ^65^Cu values and measurement precision obtained by SN-MC-ICP-MS is indicated by solid black lines and shaded areas.

The δ^65^Cu values and measurement precisions (2 s.d.) of SSC-1, SSC-3, SSC-4 and CUPD-1 determined by LA-MC-ICP-MS using each of the other three reference materials as a calibration standard are summarized in Figures 6A–D; Tables 3, 5.

**TABLE 5:** δ^65^Cu_SRM976_ values of the reference materials SSC-1, SSC-3, SSC-4 and CUPD-1 determined by LA-MC-ICP-MS.

Reference Materials	δ^65^Cu_SRM976_ (‰)	*U (‰, k = 2)* ^a^	2 s.d. (‰)^b^	n	Calibration Standards	Sources of Materials
SSC-1	0.03	0.09	0.07	132	SSC-3, SSC-4, CUPD-1	CANMET
SSC-3	0.05	0.09	0.07	154	SSC-1, SSC-4, CUPD-1	CANMET
SSC-4	0.03	0.09	0.08	144	SSC-1, SSC-3, CUPD-1	CANMET
CUPD-1	2.14	0.10	0.09	106	SSC-1, SSC-3, SSC-4	CANMET

^a^ Combined measurement uncertainty, coverage factor *k* = 2 produces an interval having a level of confidence of approximately 95%.

^b^ Precision is given as 2 standard deviation of the repeated measurements.

**FIGURE 7:**
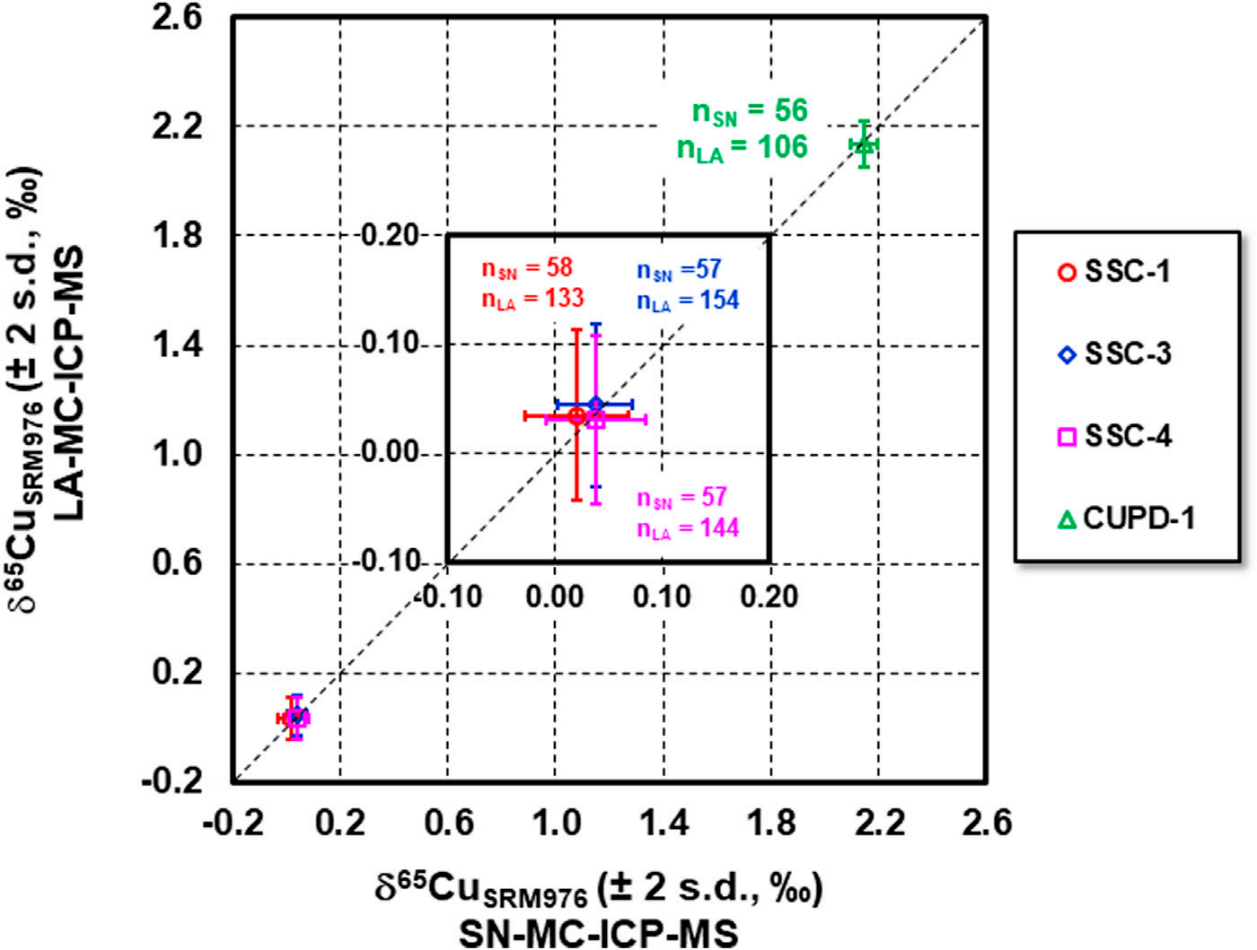
Cu isotope compositions of SSC-1, SSC-3, SSC-4 and CUPD-1 determined by LA-MC-ICP-MS vs. SN-MC-ICP-MS. The Cu isotope data overlap well the 1:1 relationship within the uncertainty, indicating that the measured Cu isotope composition of SSC-1, SSC-3, SSC-4 and CUPD-1 by LA analysis agree with those by SN analysis within the analytical uncertainties, and that precise and accurate Cu isotope measurements by LA-MC-ICP-MS using these reference materials as calibration standards were achieved.

The LA Cu isotope data for SSC-1, SSC-3, SSC-4 and CUPD-1 are plotted against the SN Cu isotope data in Figure 7. The Cu isotope data overlap, within measurement precision (2 s.d.), a 1:1 reference line, indicating that precise and accurate Cu isotope measurements by LA-MC-ICP-MS were achieved using these reference materials as calibration standards. The achievable external measurement precision (2 s.d.) for our ns pulse LA-MC-ICP-MS, expressed as relative Cu isotope amount ratios, was ± 0.07–0.09‰. This is comparable to the reported external precision of better than 0.1‰ by Jackson and Günther (2003) and Hirata et al. (2003), using ns pulse LA analysis, and Lazarov and Horn (2015) using fs pulse LA analysis with similar spot size. The measurement precision (2 standard error, number of cycles = 121) for a single measurement was 0.07‰ for ∼110 s ablation acquisition time, which is 0.035–0.055‰ higher than the reported uncertainty using fs LA analysis (Lazarov and Horn, 2015). The mean Cu signal intensity for ns pulse LA Cu isotope measurement in this study was ∼ 12 V and the calculated Poisson counting statistics error on the ^65^Cu/^63^Cu ratio was 0.000004, which is ca. 1.4 times higher than that for fs pulse LA Cu isotope measurements reported by Lazarov and Horn (2015). In our ns LA study, ca. 26% of the overall uncertainty of the ^65^Cu/^63^Cu ratio came from counting statistics, while ca. 33% of the overall uncertainty was due to counting statistics in the fs LA study of Lazarov and Horn (2015). It is worth noting that within-run measurement precision obtained using ns pulse LA Cu isotope measurement in this study was a combined uncertainty, including the uncertainties from analyses of both bracketing calibration standard and the NIST SRM976 normalization standard. Uncertainties propagating from the normalizing standard resulted in degraded analytical precision. NIST SRM976 was used both for calibration and normalization in the fs pulse LA Cu isotope study (Lazarov and Horn, 2015).

**FIGURE 8:**
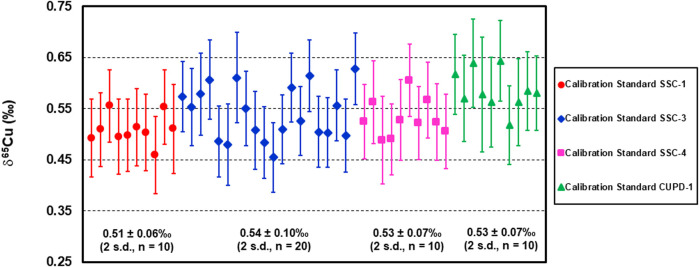
Cu isotope composition of native copper sample NMC 12864 determined by LA-MC-ICP-MS using reference materials SSC-1, SSC-3, SSC-4 and CUPD-1 as calibration standards.

Finally, the developed Cu isotope reference materials SSC-1, SSC-3, SSC-4 and CUPD-1 were employed as calibration standards in the measurement of the Cu isotope composition of a native copper sample, NMC 12864, by LA analysis. The results (Figure 8 and Supplementary Table S2) show that the mean Cu isotope composition of sample NMC 12864 was 0.54 ± 0.11‰ (*U*, *k* = 2, *n* = 50), and the Cu isotope ratios are remarkably consistent within the sample fragment (∼ 6 × 6 mm). This demonstrates that consistent Cu isotope ratio measurements were achieved using each of the developed Cu isotope reference materials as a calibration standard for LA-MC-ICP-MS Cu isotope analysis of native copper.

## Conclusions

The new Cu isotope reference materials SSC-1, SSC-3, SSC-4 and CUPD-1 can be considered isotopically homogeneous at a spatial resolution of 43 µm with 95% confidence. The Cu isotopic compositions (δ^65^Cu) and measurement precisions of SSC-1, SSC-3, SSC-4, and CUPD-1, relative to Cu isotope standard NIST SRM976, are summarized in Tables 2, 5. The Cu isotope ratios for each of the reference materials determined by LA-MC-ICP-MS are in agreement with the SN-MC-ICP-MS data within analytical uncertainty. The achievable combined measurement uncertainty of our ns pulse LA-MC-ICP-MS Cu isotope analysis of native copper (*U*, *k* = 2) was 0.09–0.10‰, whereas it was 0.04–0.08‰ by SN-MC-ICP-MS analysis. The Cu isotope composition of SSC-1, SSC-3, and SSC-4 are identical to each other within analytical uncertainty, and they are only 0.03–0.05‰ heavier than the NIST SRM976. On the other hand, CUPD-1 displays a much heavier Cu isotope composition (2.14‰) than the NIST SRM976. The unique Cu isotope composition of CUPD-1 makes it more useful in LA Cu isotope measurements of those native copper samples that exhibit similar Cu isotope compositions; for example, Bornhorst and Mathur (2017) have reported a δ^65^Cu value of 2.29‰ for a native Cu sample, which they attribute to near-surface supergene processes.

The results of this study demonstrate that SSC-1, SSC-3, SSC-4 and CUPD-1 are suitable reference materials for calibration and quality control of *in situ* LA-MC-ICP-MS Cu isotope analysis at spot size of 43 μm. These reference materials are also suitable reference materials for calibration and quality control of SN-MC-ICP-MS Cu isotope analysis. Further investigation of the homogeneity of CUPD-1 might be needed as a result of the smaller number of analyses of this sample. In addition, the influence of laser focus position along with the crater geometry should be investigated in the future for better understanding the causes of the inconsistency and relatively large variations in Cu isotope measurements for some analyses by ns pulse LA-MC-ICP-MS.

## Data Availability

The original contributions presented in the study are included in the article/Supplementary Material, further inquiries can be directed to the corresponding authors.

## Footnotes

1 CUPD-1 was discontinued after submission of this manuscript. Please contact the authors regarding the distribution of the reference materials.
