# Supplementary material for: Characterization of Four Copper Materials for Application as Reference Materials for High Precision Copper Isotope Analysis by Laser Ablation Inductively Coupled Plasma Multi-Collector Mass Spectrometry
Source: Front Chem. 2021 Apr 15;9:617205. doi: 10.3389/fchem.2021.617205 (PMC8082442; doi:10.3389/fchem.2021.617205)
Supplement: Supplementary file 1 [file datasheet1.pdf]

Supplementary Table 1 δ^{65}Cu values of reference materials ERM®-AE647 and Romil determined by SN-MC-IC-MS using each of SSC-1, SSC-3, SSC-4 and CUPD-1 as a calibration standard.

Reference Materials	Calibration Standards	$\delta^{65/63}Cu_{SRM976}$ (‰)	U (‰, k = 2)[a]	2 s.d. (‰)[b]	n	Sources
ERM®-AE647	SSC-1	0.18	0.08	0.06	19	This study
	SSC-3	0.17	0.09	0.07	12	
	SSC-4	0.19	0.08	0.07	19	
	CUPD-1	0.18	0.07	0.05	19	
		0.21		0.04	60	Moeller et al. (2012)
Romil Cu	SSC-1	0.19	0.07	0.06	16	This study
	SSC-3	0.17	0.08	0.07	17	
	SSC-4	0.18	0.07	0.07	19	
	CUPD-1	0.16	0.07	0.06	19	
		0.18		0.06	19	Moeller et al. (2012)

[a]: Combined measurement uncertainty, coverage factor k = 2 produces an interval having a level of confidence of approximately 95 percent; [b]: precision is given as 2 standard deviation of the repeated measurements.

Supplementary Table 2 $\delta^{65}Cu$ values of native copper sample NMC 12864 determined by LA-MC-IC-MS using each of SSC-1, SSC-3, SSC-4 and CUPD-1 as a calibration standard.

Sample Name	Calibration Standards	$\delta^{65/63}Cu_{SRM976}$ (‰)	U (‰, k = 2)[a]	2 s.d. (‰)[b]	n
NMC 12864	SSC-1	0.51	0.07	0.06	10
	SSC-3	0.54	0.12	0.10	20
	SSC-4	0.53	0.09	0.07	10
	CUPD-1	0.59	0.09	0.08	10

[a]: Combined measurement uncertainty, coverage factor k = 2 produces an interval having a level of confidence of approximately 95 percent; [b]: precision is given as 2 standard deviation of the repeated measurements.
