# Supplementary figures and images for: Characterization of Four Copper Materials for Application as Reference Materials for High Precision Copper Isotope Analysis by Laser Ablation Inductively Coupled Plasma Multi-Collector Mass Spectrometry
Source: Front Chem. 2021 Apr 15;9:617205. doi: 10.3389/fchem.2021.617205 (PMC8082442; doi:10.3389/fchem.2021.617205)

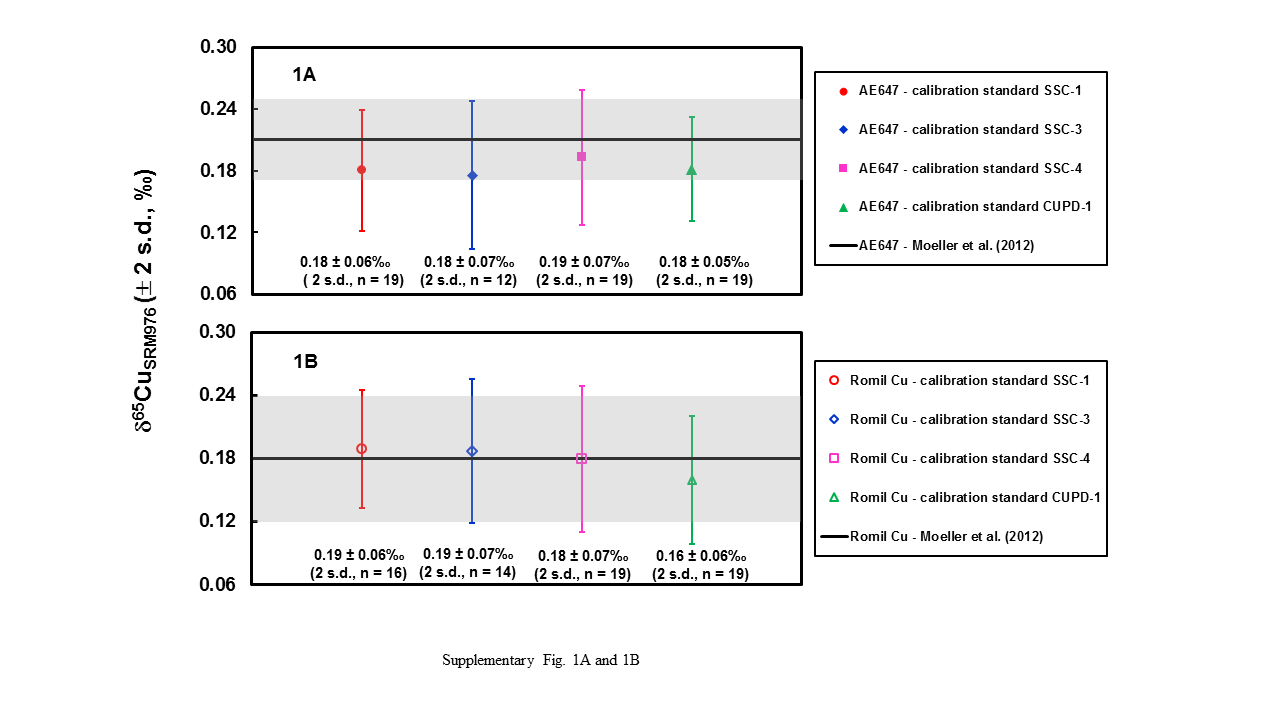

Supplement: Supplementary file 2 [file image1.tif]
